# KRAS-driven miR-29b expression is required for tumor suppressor gene silencing

**DOI:** 10.18632/oncotarget.20364

**Published:** 2017-08-19

**Authors:** Shilpa Thakur, Charles Brenner

**Affiliations:** ^1^ Department of Biochemistry, Carver College of Medicine, University of Iowa, Iowa City, IA 52242, USA

**Keywords:** DNA methylation, DNMT1, TET1, mir-29b, tumor suppressor gene

## Abstract

KRAS activation drives DNA methylation and silencing of specific tumor suppressor genes (TSGs). We previously showed that the ERK pathway induces transcriptional repression of TET1, which results in conversion of TSG promoters from a hydroxymethylated, active state to a hypermethylated and silenced state. Here we identified miR-29b as a KRAS-induced molecule that represses TET1 expression. In KRAS-transformed cells, ectopic miR-29b inhibition restores expression of TET1, thereby reactivating TSGs by reducing methylation and restoring hydroxymethylation. Mining gene expression data of lung cancer cell lines identified additional TSGs suppressed by KRAS signaling whose expression was restored by inhibition of miR-29b and re-expression of TET1. Because KRAS changes TSG promoters from hydroxymethylated to hypermethylated with miR-29b-dependent silencing of TET1, we demonstrate a model in which DNMT1 is present on target promoters prior to KRAS transformation. In addition, we propose miR-29b as a potential circulating biomarker and target for rational treatment of specific malignancies.

## INTRODUCTION

KRAS mutations are among the most common alterations in human malignancies [1–3]. The KRAS pathway turns on proliferative signals and turns off pro-apoptotic signals, thereby driving cellular transformation such that the presence of oncogenic mutations in KRAS, EGFR and other genes alters signaling pathways and gene expression programs that control responses to particular therapies [4, 5]. Moreover, because sporadic malignancies are heterogeneous, understanding the molecular differences among cancer subtypes is required to develop precision therapies. This is the central challenge of molecular oncology.

Cellular transformation is a complex process involving activation of oncogenes and silencing of tumor suppressor genes (TSGs) [6]. Chromatin alterations are common hallmarks of cancer development and progression and are frequently linked to regulation of gene expression [7]. DNA methylation is among the best characterized epigenetic alteration linked to transcriptional silencing of TSGs in KRAS-mutated cancers [8–10]. Methylation of CpG dinucleotides in DNA is a dynamically regulated process that involves cytosine 5-methylation mediated by DNA methyltransferases (DNMT1, DNMT3a, DNMT3b) [11] and active DNA demethylation initiated by the 5-mCpG hydroxylation activities of Ten-Eleven translocases (TET1, TET2, TET3) [12]. Dysregulation of DNMT and TET function is widespread in cancer especially with respect to TSG silencing [13–18].

According to a highly influential model, KRAS-induced DNMT1 transcription and resulting DNMT1 chromatin occupancy is the underlying cause of promoter hypermethylation and epigenetic silencing of multiple TSGs including *FAS* [8–10]. However, we discovered that KRAS transformation does not always transcriptionally induce DNMT1 when TSGs are hypermethylated and that KRAS-dependent suppression of TET1 is required for epigenetic silencing of TSGs [19]. Though we demonstrated that the ERK arm of the KRAS signaling pathway is responsible for TET1 repression, it was not clear how KRAS represses TET1 expression [19].

microRNAs (miRs) are short non-coding RNAs (20-30 nucleotides in length) that negatively regulate mRNA gene expression by targeting 3’-UTR sites [20]. miRNAs regulate diverse processes including cellular proliferation, differentiation and apoptosis and have been reported to function both as oncogenes and TSGs [21–23]. Oncogenic miRs can additionally be considered biomarkers associated with treatment options or emerge as cancer targets themselves [24].

Here we utilized pharmacogenomic approaches to identify miR-29b as a TET1- targeting miRNA that is upregulated by ERK activity. Inhibition of miR-29b restores *TET1* expression without affecting *DNMT1* levels. Moreover, knocking down miR-29b reactivates an array of TSGs that we found to be silenced by KRAS transformation. We further showed that an increase in *TET1* promoter occupancy and 5-hmC levels restores the epigenetic status and expression of targeted TSGs. Contrary to the expectation of the classical model of KRAS-driven DNMT1 expression [8–10], we established the presence of DNMT1 on TSGs promoters prior to oncogenic KRAS transformation with no change in DNMT1 occupancy following transformation. These mechanistic insights into reversible TSG hypermethylation in a pathway frequently altered in human cancer suggest a strategy for rational antagonism of miR-29b in tumors marked by high levels of miR-29b and low levels of TET1.

## RESULTS

### KRAS mutation induces miR-29b in an ERK-dependent manner

**Figure 1:**
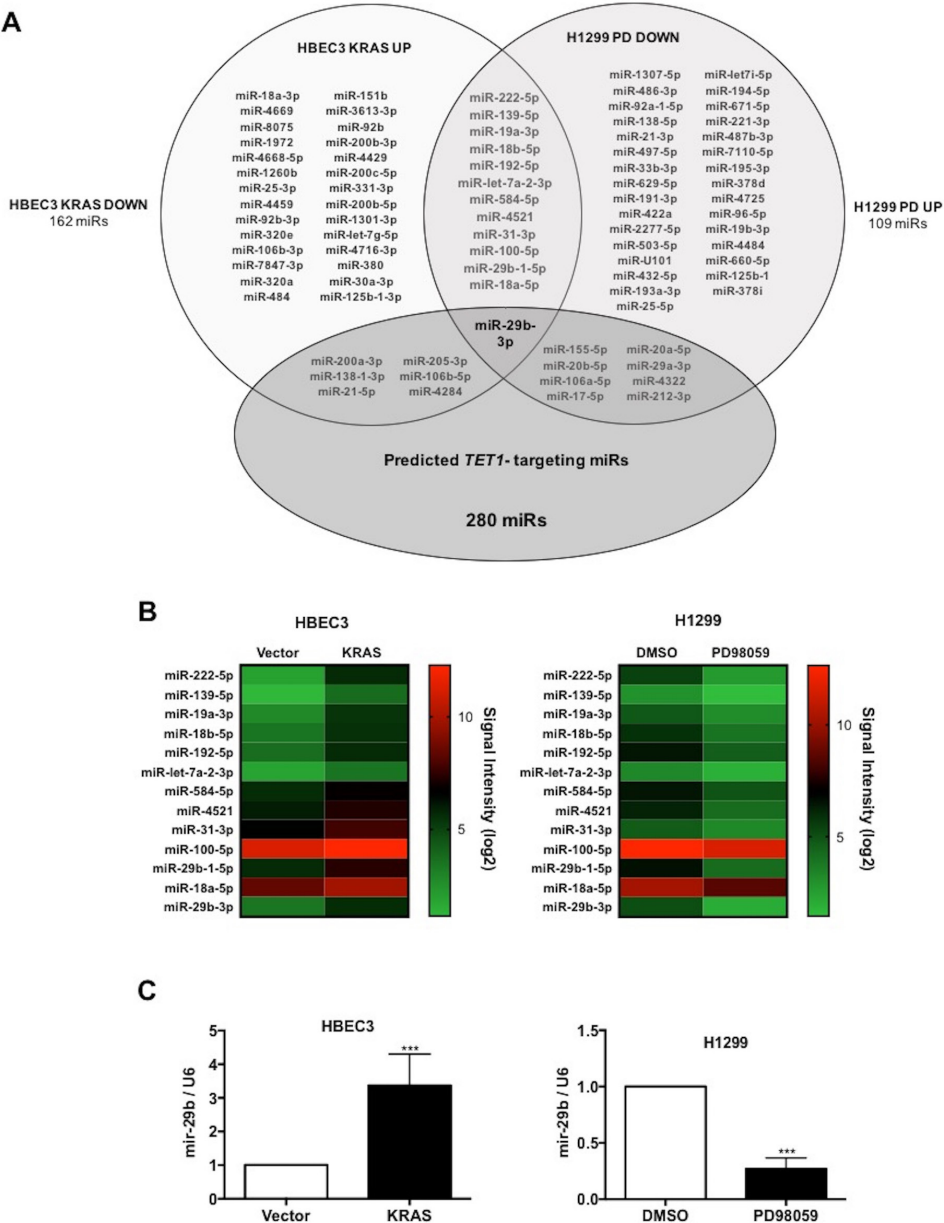
Pharmacogenomic discovery of miR-29b as a *TET1*-targeting microRNA **(A)** A Venn diagram summarizes identification of miR-29b as a predicted *TET1*-targeting microRNA whose expression depends on KRAS and MEK. **(B)** Hierarchical clustering analysis of miRNAs that depend on KRAS in HBEC3 and MEK in H1299 cells. **(C)** Validation of miR-29b expression in vector versus KRAS-transfected HBEC3 cells and DMSO versus PD98059-treated H1299 cells by qRT-PCR analysis. Data are presented as mean ± SD. ***p < 0.001 in comparison to control cells.

We previously discovered that KRAS-mediated TSG hypermethylation and silencing depends on down-regulation of the *TET1* mRNA. Suppression of *TET1* expression is mediated by the RAF-MEK-ERK pathway and not the PI3K-AKT pathway [19]. Here we aimed to identify the missing link between increased ERK activity and *TET1* suppression. As miRs are important regulators of signaling pathways in carcinogenesis, we hypothesized that a miR is up-regulated in KRAS-transformed cells that depresses expression of *TET1*. To identify miRs with the potential to regulate *TET1* that are induced by KRAS mutation in a MEK-dependent manner, we performed miR profiling with two cell lines. In HBEC3 cells, we identified the set of miRs that are up-regulated in stably KRAS-G12V transduced HBEC3 cells with respect to vector control. In addition, in KRAS-addicted H1299 cells, a MEK inhibitor PD98059 (20μM) was used to identify miRNAs downregulated by inhibition of KRAS-MEK-ERK signaling pathway with respect to a DMSO control. This approach led to identification of microRNAs which are commonly regulated by KRAS in two distinct cell lines. Among 6631 miRs analyzed, 47 are upregulated on KRAS transformation in HBEC3 cells and 53 are downregulated on PD98059 treatment in H1299 cells. Only 13 miRs were found to be commonly regulated in both cell lines (Figure 1A-1B). We further screened all miRs that were up in KRAS-transformed cells and down in PD98059-treated cells for the potential to target *TET1* mRNA using the microT-CDS prediction algorithm [25]. Though seven miRs upregulated by KRAS and 9 miRs downregulated by PD98059 were predicted to target *TET1*, miR-29b-3p was the only miR whose expression fulfilled all expression and predicted targeting criteria (Figure 1A).

To validate the microarray results, miR-29b expression was analyzed in HBEC3 and H1299 cell systems by quantitative RT-PCR. Consistent with transcriptomic analysis, KRAS transformation of HBEC3 leads to a 3-fold increased expression of miR-29b, while miR-29b is depressed nearly 4-fold by virtue of inhibition of MEK in H1299 cells (Figure 1C).

### miR-29b induction represses TET1 expression and hydroxymethylation

**Figure 2:**
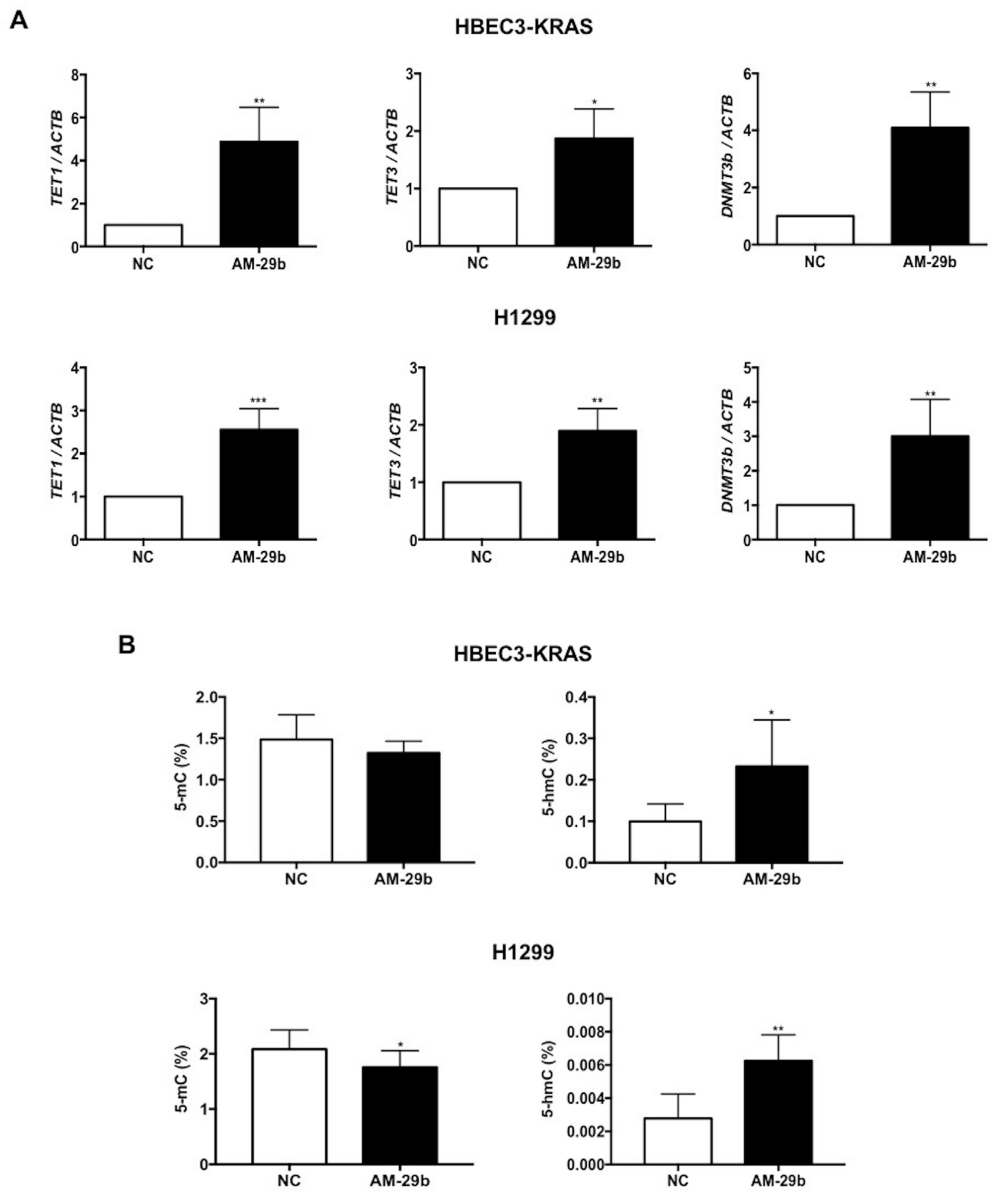
miR-29b antagonism restores *TET1* expression in KRAS-transformed cell lines **(A)** AM-29b restores expression of *TET1*, *TET3* and *DNMT3b* mRNAs in HBEC3-KRAS and H1299 cells and normalized to NC. **(B)** AM-29b decreases global 5-mC levels in H1299 cells while 5-hmC levels were significantly elevated in both cell lines upon miR-29b inhibition. Data are presented as mean ± SD. *p < 0.05; **p < 0.01; ***p < 0.001 in comparison to NC cells.

miR-29b belongs to a class of miRs reported to target epigenetic modifiers including DNMTs and TETs [26]. Induction of miR-29b by KRAS and the MAPK pathway was surprising in light of reports that miR-29b functions as a TSG, whose downregulation stimulates aberrant DNMT expression and carcinogenesis [27–31]. We therefore analyzed mRNA expression of methylating (*DNMT1, DNMT3a, DNMT3b*) and demethylating enzymes *(TET1, TET2, TET3)* as a function of antagomir-29b (AM-29b) treatment versus a negative control (NC) reagent. miR-29b expression was inhibited by 300 nM AM-29b in HBEC3-KRAS and H1299 cells (Supplementary Figure 1). We previously reported that KRAS transformation depresses expression of *TET1* and *DNMT3b* [19]. In HBEC3-KRAS cells, AM-29b restored expression of *TET1, TET3* and *DNMT3b* by 5-fold, 2-fold and 4-fold, respectively. Similar results were observed with H1299 cells (Figure 2A). AM-29b did not significantly alter expression of *TET2, DNMT1* or *DNMT3a* (Supplementary Figure 1).

Given the changes in expression of *TET1, TET3* and *DNMT3b* upon AM-29b treatment, we next investigated genome-wide 5-mC and 5-hmC levels in HBEC3-KRAS and H1299 cells. miR-29b downregulation resulted in a small but significant decrease in 5-mC levels in H1299 cells but not in HBEC3-KRAS cells. However, 5-hmC levels were significantly elevated in both cell lines upon miR-29b inhibition, indicating an overall increase in TET activity (Figure 2B).

### Oncogenic miR-29b induction causes repression of lung TSGs

**Figure 3:**
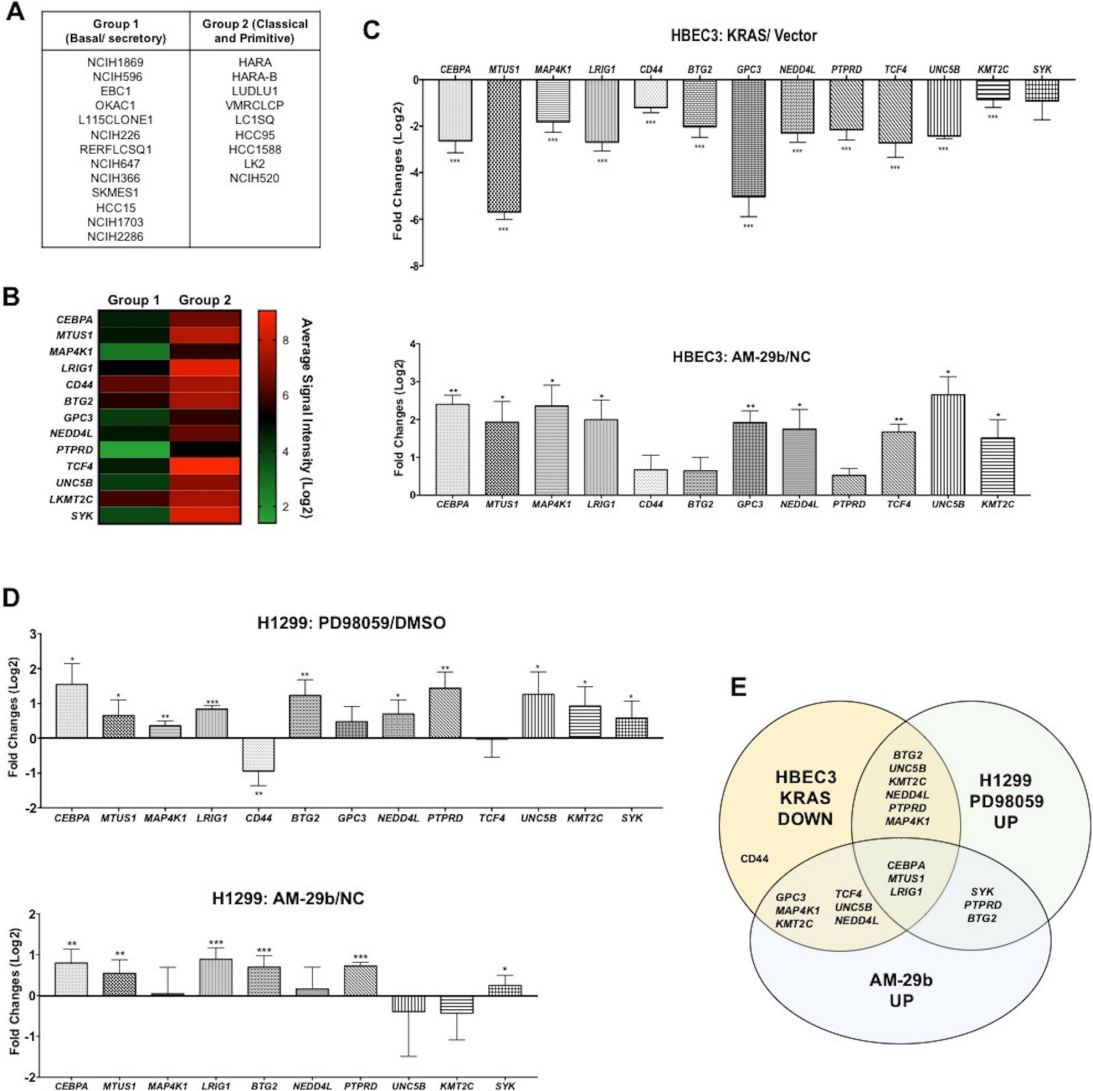
miR-29b dependent transcriptional suppression of TSGs downstream of KRAS transformation **(A)** Classification of lung cancer cell lines [4]. **(B)** Hierarchical clustering analysis of TSGs, whose mRNAs are depressed >1 log fold- change in Group 1 cell lines with respect to Group 2. **(C)** TSGs are consistently depressed in KRAS-transformed HBEC3 cells with respect to controls (upper panel). The same genes are re-expressed upon AM-29b transfection (lower panel). **(D)** Bioinformatically identified genes are almost universally reactivated by PD98059 and AM-29b in H1299 cancer cells. **(E)** A Venn diagram depicts the high overlap of TSGs silenced by KRAS transformation, reactivated by PD98059 and restored by AM-29b. Data are presented as mean ± SD. *p < 0.05; **p < 0.01; ***p < 0.001 in comparison to control cells.

Lung squamous cell carcinoma (SCC) has been classified into three distinct subtypes based on gene enrichment profiles: basal/secretory, classical and primitive [4]. The basal/secretory subtype, also termed an immune evasion subtype, is enriched in MAPK signaling with miR-29b induction and *TET1* downregulation. To identify additional genes that are coordinately regulated by KRAS, ERK, miR-29b and TET1, we used GEO2R (http://www.ncbi.nlm.nih.gov/geo/geo2r/?acc=GSE57083) to mine expression data of 13 basal/secretory SCC cell lines versus 9 cell lines of the classical or primitive subtypes (Figure 3A). Focusing on TSGs, we analyzed expression of a set of 534 mRNAs depressed in lung SCC compared to normal lung in the Tumor Suppressor Gene database (https://bioinfo.uth.edu/TSGene/). As shown in Supplementary Table 1, we identified 44 genes that are significantly down-regulated in the basal/secretory subtype. For further validation in HBEC3 and H1299 cells, we selected 13 genes with more than one log fold-change of down-regulation in the basal/secretory SCC lines (Figure 3B).

As shown in Figure 3C, nearly all of these genes are down-regulated by KRAS-transformation in HBEC3 cells and restored by AM-29b. Similarly, most are increased in expression by PD95059 and AM-29b in H1299 (Figure 3D). Thus, miR-29b is an important mediator of KRAS-dependent TSG silencing in human basal/secretory lung cancer.

### miR-29b inhibition reverses hypermethylation-mediated silencing of *MGMT* and *DAPK* genes in KRAS-transformed cells

**Figure 4:**
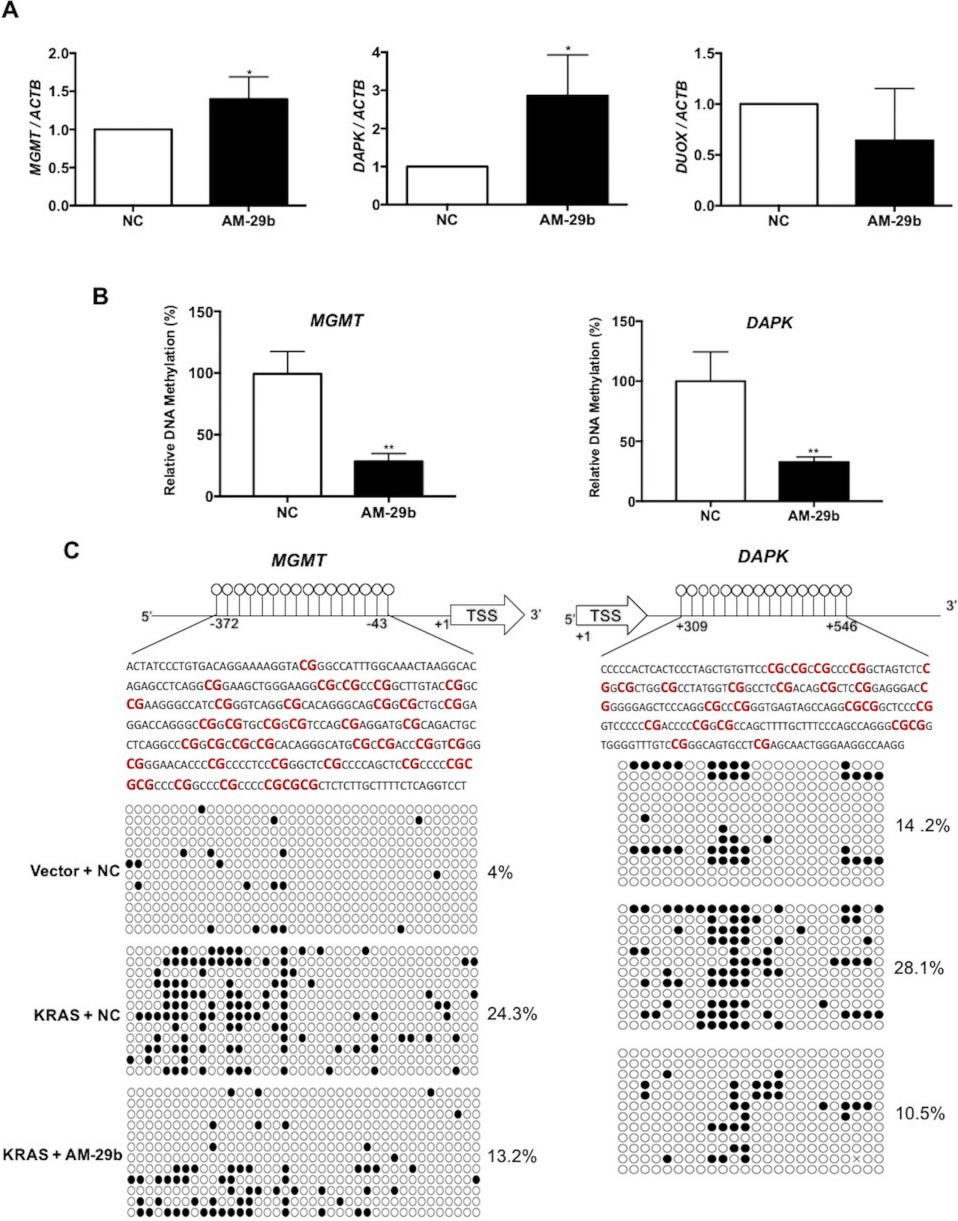
Blocking miR-29b restores the methylation status of *DAPK* and *MGMT* in AM-29b treated HBEC3-KRAS cells **(A)** AM-29b reactivates expression of the *MGMT* and *DAPK* TSGs. **(B)** AM-29b reduces hypermethylation of the *MGMT* and *DAPK* promoters. **(C)** AM-29b reverts specific KRAS-induced hypermethylation of *MGMT* and *DAPK* CpG islands. Nonmethylated and methylated CpGs are depicted as open and solid circles, respectively. Data are presented as mean ± SD. *p < 0.05; **p < 0.01 in comparison to control cells.

We previously reported that epigenetic silencing of three TSGs (*DAPK, MGMT* and *DUOX1*) caused by KRAS-driven promoter hypermethylation is a function of repressed expression of *TET1* [19]. Identification of miR-29b as a factor that depresses *TET1* expression in KRAS-transformed cells suggested the possibility of reversing TSG silencing with a drug modeled after AM-29b. To test the cellular basis of this hypothesis, HBEC3-KRAS cells were transfected with AM-29b and the mRNA levels of *MGMT, DAPK* and *DUOX1* were analyzed. As shown in Figure 4A, miR-29b inhibition restores mRNA accumulation of *MGMT* and *DAPK* without affecting steady-state levels of *DUOX1*. To test whether a decrease in promoter methylation is responsible for restored gene expression, we examined the promoter methylation status of *MGMT* and *DAPK* genes by quantitative methylated DNA immunoprecipitation (MeDIP). As shown in Figure 4B, AM-29b treatment produces a significant decrease in *MGMT* and *DAPK* promoter methylation, indicating that miR-29b drives reversible TSG silencing via increasing net DNA methylation. The results of MeDIP assay were confirmed by bisulfite sequencing. Analysis of 39 and 24 CpG sites in the *MGMT* and *DAPK* promoters revealed a 1.8- and 2.7-fold decrease in promoter methylation following AM-29b treatment in KRAS-transformed cells, respectively (Figure 4C). Together, our data indicate that KRAS-directed epigenetic silencing of *MGMT* and *DAPK* occurs *via* a hypermethylation mechanism that can be reversed by miR-29b inhibition.

### Reduction in TET1-mediated DNA demethylation is responsible for increased promoter methylation

**Figure 5:**
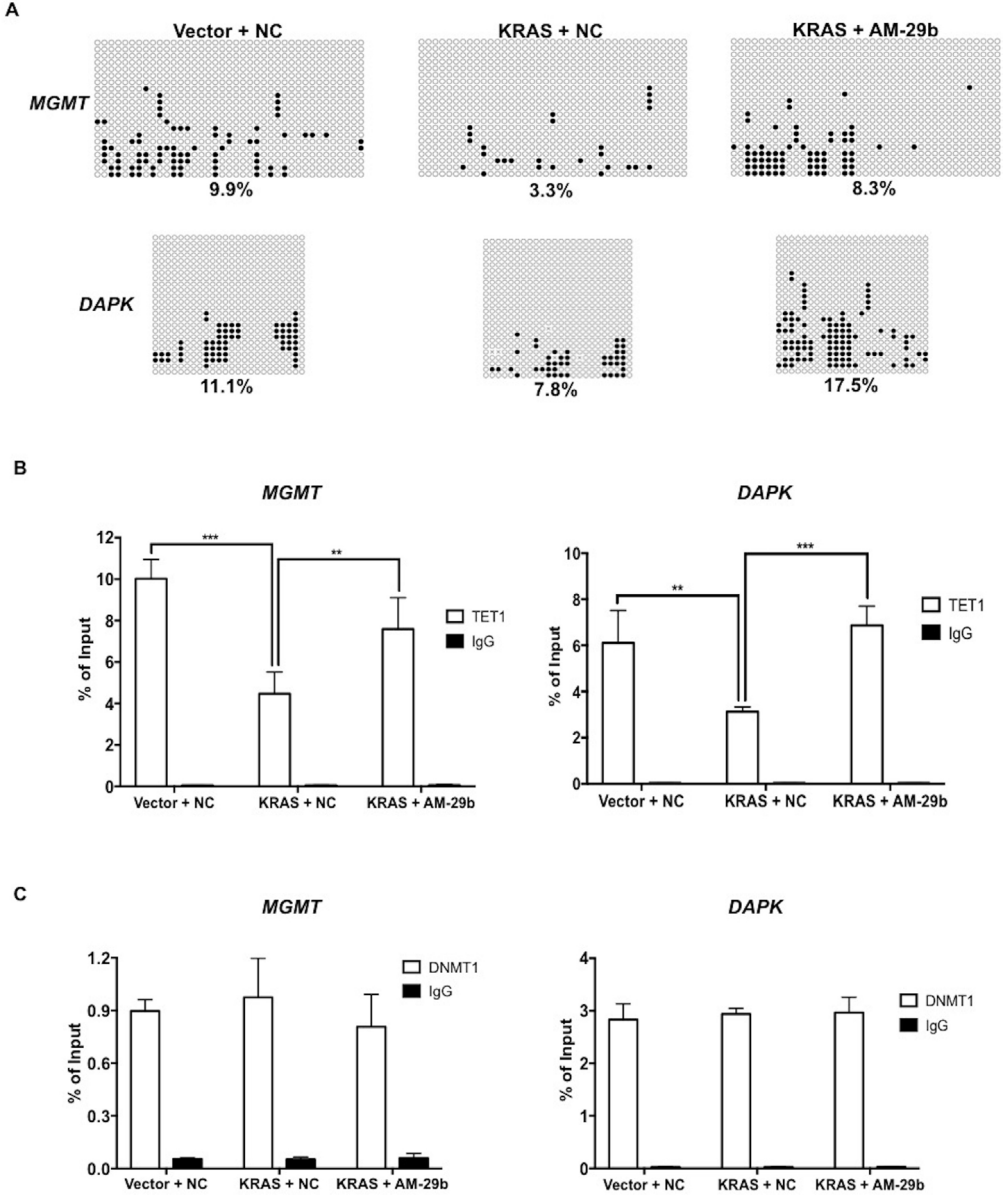
RAS and miR-29b-controlled TET1 chromatin occupancy controls the epigenetic status of *MGMT* and *DAPK* **(A)** KRAS transformation depresses and miR-29b antagonism restores the 5-hmC status of *MGMT* and *DAPK* promoters. Open circles represent 5mC and C, filled circles represent 5-hmC, and X marks indeterminant sites. **(B)** KRAS transformation depresses and miR-29b antagonism restores TET1 occupancy of the *MGMT* and *DAPK* promoters. **(C)** In contrast to gene expression and 5-mC status which are regulated by KRAS and miR-29b, DNMT1 occupancy of *MGMT* and *DAPK* promoters is not regulated by KRAS or miR-29b. Data are presented as mean ± SD. **p < 0.01; ***p < 0.001 in pairwise comparisons.

We and others have established that decreased TET1 expression in response to oncogenic KRAS, MAPK or EGFR signaling is responsible for TSG silencing [4, 19, 32]. However, the standard model of KRAS-induced hypermethylation emphasizes the role of induced expression of DNMT1 as the driver of this phenomenon [8]. To test whether demethylation induced by miR-29b inhibition is caused by restored TET1 activity, we quantified 5-hmC modifications in the *MGMT* and *DAPK* promoters with TET-assisted bisulfite sequencing (TAB-seq). As shown in Figure 5A, KRAS transformation produces a decrease in promoter 5-hmC modifications and AM-29b treatment reverts this effect. The extent of 5-hmC modifications was more than doubled from 3.3% to 8.3% and 7.8% to 17.5% in the *MGMT* and *DAPK* promoters upon miR-29b inhibition, respectively. To test the hypothesis that KRAS-driven methylation of these genes is caused by decreased TET1 binding to their promoters, TET1 chromatin immunoprecipitation (ChIP) was performed. As shown in Figure 5B, KRAS transformation clearly depresses TET1 occupancy of these promoters, which was restored by AM-29b treatment.

The standard model of KRAS-driven TSG silencing states that DNMT1 and other RAS epigenetic silencing factors (RESEs) are not present on target gene promoters prior to KRAS transformation [8]. However, our data indicated that target gene promoters are enriched in *5-hmC prior to KRAS transformation* and that DNMT1 expression is not induced by KRAS in systems that nonetheless exhibit KRAS-driven TSG methylation [19]. We reasoned that the TET product 5-hmC cannot be present if the TET1 substrate 5-mC is not there first. According to this view, genes subject to KRAS-driven TSG methylation are dually occupied by DNMT1 and TET1 prior to activation of the MAPK pathway. Activation of the MAPK pathway would lead to miR-29b induction and TET1 repression, leading to net DNA methylation secondary to the loss of TET1-dependent active DNA demethylation.

**Figure 6:**
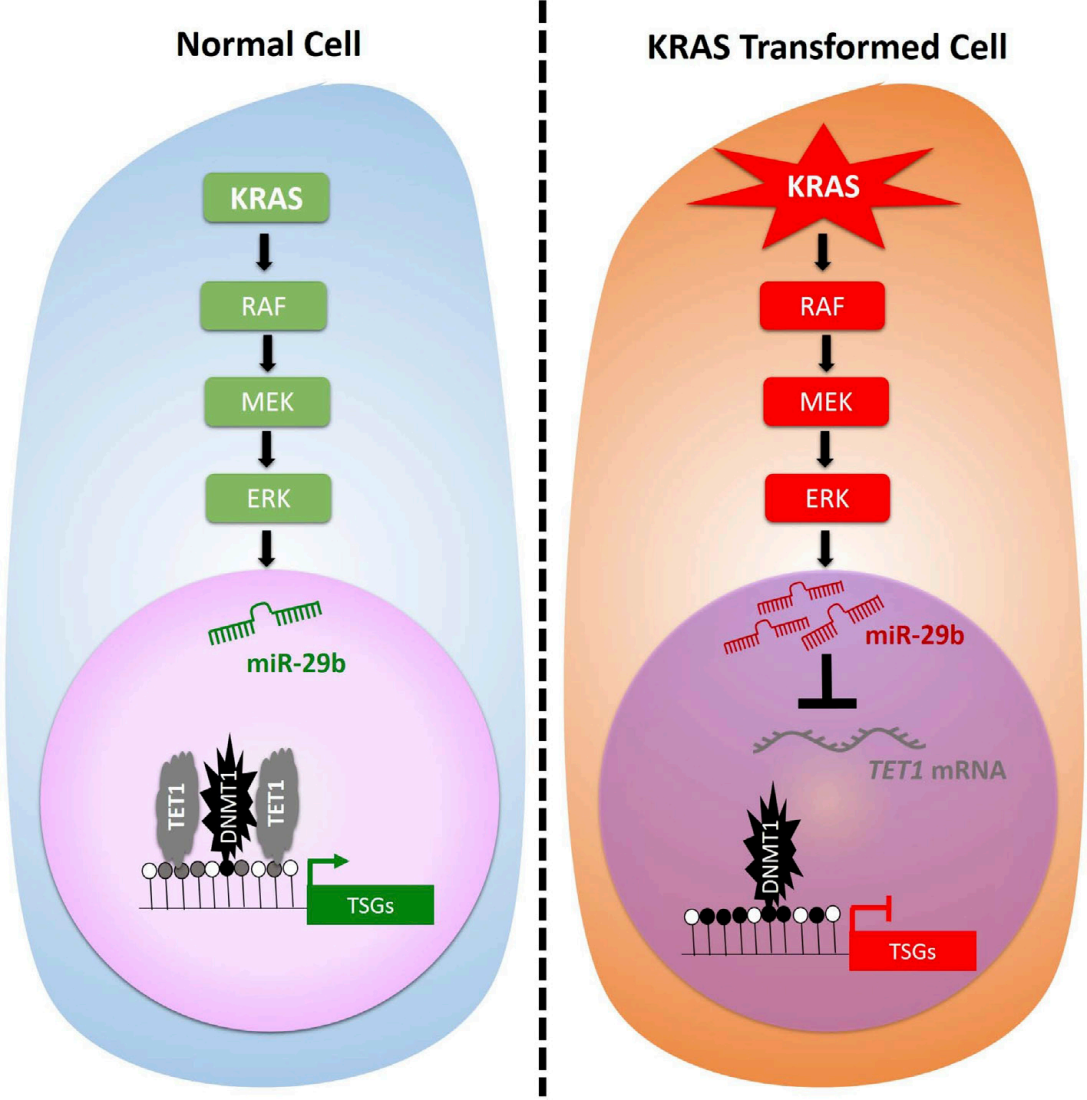
MEK-dependent miR-29b induction represses TET1 expression, thereby leading to RAS-dependent TSG hypermethylation and silencing In contrast to earlier models, which proposed that KRAS drives DNMT1 transcription leading to TSG hypermethylation, our data indicate that KRAS drives miR-29 induction through the RAF-MEK-ERK pathway and that net hypermethylation depends on down-regulation of TET1. Moreover, TET1 and DNMT1 are both present on target gene promoters prior to KRAS activation.

As shown previously, KRAS transformation does not alter expression of *DNMT1* or *DNMT3a* in HBEC3 or H1299 cells [19], while *DNMT3b* expression is depressed by *KRAS* transformation and restored by miR-29b inhibition (Figure 2A). To test the hypothesis that DNMT1 is already present on KRAS-, miR-29b- and TET1-regulated promoters, we performed DNMT1 ChIP. Whereas TET1 is responsive to KRAS transformation and miR-29b antagonism (Figure 5B), DNMT1 is not: it is simply present on KRAS-regulated promoters. Thus, we demonstrated not only that miR-29b mediates KRAS-driven TSG silencing but that net methylation of *MGMT* and *DAPK* promoters is due to evacuation of TET1 from promoters that have DNMT1 and TET1 present prior to KRAS activation. The results are graphically summarized in Figure 6.

## DISCUSSION

Lung cancers kill more people than tumors initiating in any other organ system [33, 34]. Most such malignancies occur after years of tobacco carcinogenesis and involve many altered genes [35, 36]. EGFR and KRAS mutations are the most common and mutually exclusive mutations in malignancies of the lung [37, 38]. Because KRAS is an effector of EGFR, the EGFR-RAS-RAF-MEK-ERK cascade is considered a target-rich environment for medical management of lung cancer [37, 39–41]. Whereas genetic alterations reveal oncoprotein targets, we also appreciate that the same signaling pathway turns off an abundance of TSGs via gene silencing. Nucleoside-based DNA demethylating agents, such as 5-aza-cytidine, reactivate hypermethylated TSGs via trapping DNMTs for subsequent proteolytic elimination [42]. Direct inhibitors of DNMT1 have recently been reported [43–45]. However, there are only limited data showing that DNMT1 can be targeted with such compounds for the prevention or treatment of cancer [46].

The concept of RESE suggested that factors in addition to DNMT1 might be targetable in malignancies with MAPK activation [8]. However, we have shown that KRAS-driven hypermethylation and gene silencing can occur without induction of the DNMT1 mRNA or protein and that the gene silencing depends on repression of TET1 [19]. This led us to search for a RESE that is downstream of MAPK activation and required for TET1 repression. Such a molecule, if antagonizable, could potentially emerge as a target to reactivate TSGs downregulated in common human malignancies [47–49].

Here we show that the KRAS and MAPK pathway induces miR-29b expression leading to *TET1* suppression and epigenetic silencing of genes such as *DAPK* and *MGMT* in KRAS-transformed lung cells. Contrary to the predictions of the classical model of KRAS-driven TSG methylation [8], these genes are occupied by DNMT1 and TET1 prior to KRAS transformation and gain net methylation due to relief of TET1-dependent hydroxymethylation. Our data indicate that miR-29b downregulates a set of TSGs that contribute to transformation by KRAS and that these genes can be identified using bioinformatic approaches.

In the basal/secretory subtype of lung SCC, the ETS1 transcription factor drives expression of miR-29b [4]. In addition, oncogenic EGFR signaling was reported to induce expression of transcriptional repressor YY1 and down-regulate expression of CEBPA in order to repress TET1 [32]. However, because we discovered that CEBPA is re-expressed upon miR-29b antagonism (Figure 3), it is not clear that CEBPA acts upstream of TET1.

Discovery of miR-29b in an oncogenic context was surprising in view of its earlier characterization as a TSG in lung [31] and other malignancies [27, 28]. Our results were particularly surprising in that miR-29b was reported to downregulate DNMT3A and DNMT3B directly [31] and DNMT1 expression indirectly [28]. However, we found no changes in *DNMT1* or *DNMT3A* expression following miR-29b inhibition. Whereas *DNMT3B* expression does increase upon miR-29b inhibition, this does not appear to be consequential to KRAS-driven TSG silencing as one would either expect DNMTs to be overexpressed when the KRAS pathway is on [8] and/or to find that DNMT-opposing TETs are repressed when the KRAS pathway is on [19]. Moreover, we are not alone in identifying miR-29b as an oncogene in lung cancer as its expression has been shown to protect KRAS-transformed lung cells from apoptosis by inducing the NF-κB pathway [5].

As shown in Figure 3, our data show that miR-29b antagonism is effective in restoring TSG expression in KRAS-activated cancer cells and identify cancer gene expression subtypes that rationalize AM-29b drug development. Naturally, in malignancies in which miR-29b is instead a TSG, miR-29b would not be a target. We suggest that the gene set enrichment methods [4] that were expanded herein be used to identify tumors that are responsive to miR-29b antagonism. Ideally, this transcriptomic analysis should include mRNAs and miRNAs so that one can see *ETS-1* and miR-29b increased with *TET1* decreased as a subtype of cancer that could be opposed by miR-29b antagonism. In addition, because tumors with inactivated miR-29b would not be positive for miR-29b in a liquid biopsy, a simple approach to identify candidates for miR-29b antagonism would be to screen for elevated circulating miR-29b and any other biomarker(s) of MAPK hyperactivity in liquid biopsies.

## MATERIALS AND METHODS

### Cell lines

Vector or KRAS-G12V transduced HBEC3 cells were cultured in keratinocyte serum-free media supplemented with bovine pituitary extract and recombinant human EGF. H1299 cells were cultured in RPMI-1640 media supplemented with 10% fetal bovine serum.

### miRNA array

miRNA profiling was performed with an Affymetrix GeneChip miRNA 4.0 array. RNA integrity number was > 9 for all samples. The Affymetrix Expression Console and Transcriptome Analysis Console software 3.0 were used to analyze raw data and generate heat maps. miRNA profiling data have been deposited to the NCBI Gene Expression Omnibus, accession number GSE100857.

### RNA isolation and real-time qPCR

Total RNA was isolated using the mirVANA miRNA isolation kit (Ambion, Life Technologies). For miRNA analysis, cDNA was synthesized using TaqMan MicroRNA Reverse Transcription Kit (ThermoFischer Scientific) and miR-29b/U6 expression was quantified using TaqMan MicroRNA Assays (ThermoFischer Scientific). mRNA expression of target genes was determined using iScript cDNA Synthesis Kit and iQ SYBR Green Supermix (Bio-Rad) on a CFX96 Real-Time PCR Detection System (Bio-Rad). Primer pairs used in mRNA expression analysis are listed in Supplementary Table 2.

### Transfection

AM-29b and NC reagents were purchased from ThermoFisher Scientific. Cells were transfected with 300 nM AM-29b or NC using the Lipofectamine RNAiMAX Reagent (Invitrogen) and harvested after 72 h.

### ChIP and MeDIP

ChIP was performed using a Pierce Magnetic ChIP Kit (ThemoFisher Scientific) as instructed. Ten percent of digested chromatin was saved as input. TET1 and DNMT1 antibodies were purchased from Active Motif (61443) and Santa Cruz Biotechnology (sc-10219), respectively. MethylMiner Methylated DNA Enrichment Kit (ThemoFisher Scientific) was used for methylation analysis at TSG promoter regions. Genomic DNA was isolated using DNeasy Blood and Tissue kit (QIAGEN) and fragmented to an average size of 400 bp using a Covaris S2 sonicator. Immunoprecipitated DNA was analyzed by qPCR using primers listed in Supplementary Table 2.

### Bisulfite and TAB sequencing

EpiTect bisulfite kit (QIAGEN) was used for bisulfite treatment of genomic DNA. Treated DNA was amplified using gene specific primers (Supplementary Table 2). PCR products were run on 2% agarose gels and purified using QIAquick Gel Extraction Kit (QIAGEN). Purified products were cloned into the pGEM-T easy vector (Promega) and individual clones were then selected for sequencing. Methylation status of individual CpGs was assessed using the QUMA tool (http://quma.cdb.riken.jp). To detect 5-hmC modifications, genomic DNA was fragmented to an average of 400 bp by sonication. To protect 5-hmC modifications, fragmented DNA was treated using a TAB-seq Kit (WiseGene). Treated DNA was then subjected to bisulfite conversion, amplification, cloning and sequencing as above.

### 5-mC and 5-hmC quantification

Genomic DNA was isolated from AM-29b or NC transfected HBEC3-KRAS and H1299 cells. One hundred nanograms of DNA was used to determine global methylation and hydroxyl methylation levels using 5-mC and Quest 5-hmC DNA ELISA Kits (Zymo Research), respectively.

### Statistical analysis

Data were analyzed using GraphPad Prism software 7.0a. p-values were calculated using two-tailed Student’s t-tests and were considered to be significant if less than 0.05. All data were presented as mean ± SD.

## SUPPLEMENTARY MATERIALS FIGURE AND TABLES


