# Supplementary material for: KRAS-driven miR-29b expression is required for tumor suppressor gene silencing
Source: Oncotarget. 2017 Aug 19;8(43):74755–66. doi: 10.18632/oncotarget.20364 (PMC5650376; doi:10.18632/oncotarget.20364)
Supplement: Supplementary file 1 [file oncotarget-08-74755-s001.pdf]

KRAS-driven miR-29b expression is required for tumor suppressor gene silencing

SUPPLEMENTARY MATERIALS

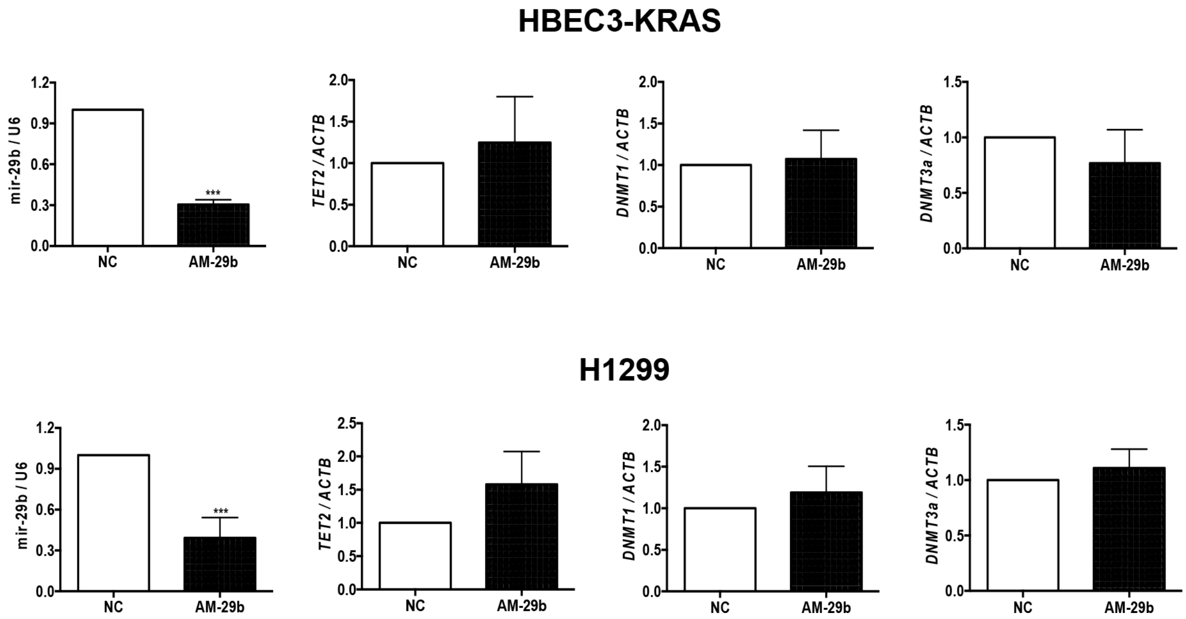

Supplementary Figure 1: miR-29b inhibition showed no significant effect on TET2, DNMT1 and DNMT3a expression, related to Figure 2A. miR-29b and mRNA levels were determined by qRT-PCR in AM-29b (300 nM) transfected HBEC3-KRAS and H1299 cells and normalized to negative control.

Supplementary Table 1: List of tumor suppressor genes downregulated in Group 1 cell lines over Group 2

Gene name	Log Fold- Change	Gene name	Log Fold- Change
MAP4K1	1.29	TSC22D1	0.27
ESR1	0.176	CD44	2.88
UNC5B	1.7	CEBPA	1.18
NAPEPLD	0.74	BTG3	0.435
LHX6	0.94	CUX1	0.94
HIPK2	0.45	ASXL1	0.86
HIVEP1	0.36	PDS5B	0.48
LRMP	0.62	TRIM35	0.51
SMAD4	0.51	FOXO1	0.93
NRF1	0.24	LRIG1	1.97
HECA	0.49	FOXP1	0.31
KDM3B	0.4	GPC3	1.77
RTN4	0.79	IGF2R	0.81
PTCH1	0.77	NOTCH1	0.47
NDRG2	0.53	PAIP2	0.57
MTUS1	2.29	ING3	0.4
PTPN13	0.59	KLK10	0.52
PTPRD	2.22	SYK	2.8
KMT2C	1.16	TCF4	3.26
NEDD4L	1.39	BTG2	1.7
TBRG1	0.55	SPOP	0.54
TNFRSF10A	0.75	PER2	0.91

Supplemenatry Table 2: List of primers related to experimental procedures

	Forward	Reverse
**mRNA**		
ACTB	AGAGGGAAATCGTGCGTGAC	CAATAGTGATGACCTGGCCGT
KRAS	TGTGGTAGTTGGAGCTGGTG	TGACCTGCTGTGTCGAGAAT
TET1	ACCCCCTGTCACCTGCTGAGG	GCGATGGCCACCCCACCAAT
TET2	TCACACCAGGTGCACTTCTC	GGATGGTTGTGTTTGTGCTG
TET3	TCTCCCCAGTCTTACCTCCG	CCAGGCTTCAGGGAACTCAG
DNMT1	GAGCTACCACGCAGACATCA	CGAGGAAGTAGAAGCGGTTG
DNMT3a	CAAGCGGGACGAGTGGCTGG	TCAGTGGGCTGCTGCACAGC
DNMT3b	CTCAGAGGCAGTGACAGCAG	TGTCTGAATTCCCGTTCTCC
DAPK	TGGAGACACTAAGCAAGA	GGCACTGGTATTACTGAAG
MGMT	ACGCACCACACTGGACAGCC	CCGGCACGGGGAACTCTTCG
DUOX1	ATGTGCCAGATACCCAAAGC	CAGCTGACGGATGACTTGAA
CEBPA	GACATCAGCGCCTACATCG	GGCTGTGCTGGAACAGGT
MTUS1	CCAATAGCGAACCACATT	TCATCATCAATAAGACAATAGGA
LRIG1	CTTATGAGTGCCAATGTG	GGAAGTAAGATTAGTCAGTTAA
CD44	TACAATAAGAGAAGAAGCCAA T	GCATCAAGAACAGACACT
BTG2	CTTAGGGAACCATCTCTC	TTCAGCCAAGGAATACAT
KMT2C	TTCTACTGGATTGATGGA	TATGTCTGCTGATGATGA
GPC3	CCTTGAAGAACTTGTGAAT	CTGGACATACTGGATAGAA
MAP4K1	CCTTCCACAACTTCATCAA	ATGACTGAGCATCTTGGT
NEDD4L	TATGCTGAAGTGAGGATT	ATTATGCTGTGAAGATGAC
PTPRD	CAACAACAATGGTCGTATT	CTCTTCACTCTGCTCAAT
SYK	GATGCTGGTTATGGAGATG	TCTATGATGTTCTTATCCTTGAC
TCF4	TGCCACATTGCTTCATTA	GTCTGCGATTCATAACTACT
UNC5B	TTCAGACCGCTAGTAAGG	CATTCTCATCCACACTCAAG
**MeDIP/ChIP**		
DAPK	GCTTTTGCTTTCCCAGCCAGGGC	ATCGCACTTCTCCCCGAAGCCAA
MGMT	GAACGCTTTGCGTCCCGACG	CCGAGGGAGAGCTCCGCACT
**Bisulfite sequencing**		
DAPK	TTTTTATTTATTTTTTAGTTGTGTTTT	CCTTAACCTTCCCAATTACTC
MGMT	ATTATTTTTGTGATAGGAAAAGGTA	AAAACCTAAAAAAAACAAAAAAAC
